# Dietary Choline and Betaine Intake and Risk of Colorectal Cancer in an Iranian Population

**DOI:** 10.3390/cancers15092557

**Published:** 2023-04-29

**Authors:** Monireh Sadat Seyyedsalehi, Marta Rossi, Maryam Hadji, Hamideh Rashidian, Maryam Marzban, Maria Parpinel, Federica Fiori, Ahmad Naghibzadeh-Tahami, Yusuf A. Hannun, Chiara Luberto, Kazem Zendehdel, Paolo Boffetta

**Affiliations:** 1 Department of Medical and Surgical Sciences, University of Bologna, 40138 Bologna, Italy; monireh.seyyedsalehi@studio.unibo.it; 2 Cancer Research Center, Cancer Institute, Tehran University of Medical Sciences, Tehran 5166614711, Iran; 3 The Department of Clinical Sciences and Community Health, Università Degli Studi di Milano, 20133 Milan, Italy; 4 Health Sciences Unit, Faculty of Social Sciences, Tampere University, 33521 Tampere, Finland; 5 Department of Human Genetics, McGill University, Montreal, QC 3640, Canada; 6 Department of Medicine, University of Udine, Via Colugna 50, 33100 Udine, Italy; 7 Modeling in Health Research Center, Institute for Futures Studies in Health, Kerman University of Medical Sciences, Kerman 7619833477, Iran; 8 Health Foresight and Innovation Research Center, Institute for Futures Studies in Health, Kerman University of Medical Sciences, Kerman 7619833477, Iran; 9 Stony Brook Cancer Center, Stony Brook University, New York, NY 11794, USA; 10 Department of Medicine, Stony Brook University, New York, NY 11794, USA; 11 Department of Physiology and Biophysics, Stony Brook University, Stony Brook, NY 11794, USA; 12 Cancer Biology Research Center, Cancer Institute, Tehran University of Medical Sciences, Tehran 5166614711, Iran

**Keywords:** choline, colorectal cancer, phosphocholine, sphingomyelin, carcinogen, betaine

## Abstract

**Simple Summary:**

Colorectal cancer (CRC) is increasing in low- and middle-income countries, likely due to changing lifestyle habits, including diet. We found that a higher total choline intake was associated with an elevated risk of CRC, as well as glycerophosphocholine (GPC) and sphingomyelin (SM). Conversely, betaine intake was associated with a decreased risk of CRC. Consequently, it is possible to focus on the intake of betaine sources and manage the use of animal products as references for SM or other choline types with risks of CRC.

**Abstract:**

Background: Colorectal cancer (CRC) is increasing in low- and middle-income countries, likely due to changing lifestyle habits, including diet. We aimed to investigate the relationship between dietary betaine, choline, and choline-containing compounds and CRC risk. Methods: We analyzed data from a case–control study, including 865 CRC cases and 3206 controls from Iran. Detailed information was collected by trained interviewers using validated questionnaires. The intake of free choline, phosphocholine (Pcho), glycerophosphocholine (GPC), phosphatidylcholine (PtdCho), and sphingomyelin (SM), as well as of betaine was estimated from food frequency questionnaires and categorized into quartiles. The odds ratios (OR) and 95% confidence intervals (CI) of CRC for choline and betaine quartiles were calculated using multivariate logistic regression by adjusting for potential confounders. Results: We observed excess risk of CRC in the highest versus lowest intake of total choline (OR = 1.23, 95% CI 1.13, 1.33), GPC (OR = 1.13, 95% CI 1.00, 1.27), and SM (OR = 1.14, 95% CI 1.01, 1.28). The intake of betaine exerted an inverse association with CRC risk (OR = 0.91, 95% CI 0.83, 0.99). There was no association between free choline, Pcho, PtdCho, and CRC. Analyses stratified by gender showed an elevated OR of CRC in men for SM intake OR = 1.20, 95% CI 1.03, 1.40) and a significantly decreased CRC risk in women for betaine intake (OR = 0.84, 95% CI 0.73, 0.97). Conclusion: Dietary modifications leading to an increase in betaine sources and managing the use of animal products as references for SM or other choline types might contribute to decreasing the risk of CRC.

## 1. Introduction

Colorectal cancer (CRC), which encompasses colon and rectum cancers, is the world’s third most commonly diagnosed cancer (1.9 million new cases) and was the second most fatal cancer (1.0 million cancer deaths) in 2020 [1,2]. However, the incidence and mortality of CRC vary considerably between countries and regions. While CRC incidence and mortality remain relatively high in high-income countries, they are increasing in low- and middle-income countries for both men and women [3]. It is predicted that the number of incident cases will increase by 54.1% in Iran, an upper- to middle-income country, between 2016 and 2025, from 11,558 to 17,812 [4].

Modifiable risk factors contributing to CRC development are related to individual habits or lifestyle, such as obesity, physical activity, and diet [5]. Choline (2-hydroxyethyl-trimethyl-ammonium salt; molecular weight of 104 g/mol) is essential for maintaining the structure and function of cells. In humans, it can be obtained either by endogenous synthesis or from dietary sources including red meat, chicken, eggs, milk and fish, and some plant foods, such as certain beans and cruciferous vegetables [6,7]. Choline is found in different specific forms in food, including free choline, phosphocholine (Pcho), glycerophosphocholine (GPC), phosphatidylcholine (PtdCho), and sphingomyelin (SM). Choline can affect four main metabolic pathways involved in the synthesis of acetylcholine, trimethylamine (TMA), betaine, and phospholipids [8]. Choline intake may act as an influential factor in folate and choline methyl donation, which have been found to have adverse effects on cancer or heart disease [9,10].

According to in vitro and epidemiological studies, as a result of its contribution to several mechanisms, such as anti-inflammatory and antioxidant mechanisms, betaine was found to have positive effects on metabolic syndrome, Alzheimer’s disease, and cancer. Aside from the body synthesizing betaine from choline, it can also be found in high levels in wheat bran, wheat germ, spinach, and beets [11,12,13].

Dietary choline and betaine have been related to CRC risk, although the evidence is still inconclusive due to the variation of choline types and CRC locations. Different dietary habits around the world may also affect the results [14,15]. To the best of our knowledge, there is not any study on dietary choline intake with a focus on different species and CRC in the EMRO region, particularly the Iranian population. To address this issue, we conducted a large study and investigated the association between CRC risk and the intake of total and specific types of choline, in particular SM, and betaine as a product of choline metabolism.

## 2. Materials and Methods

### 2.1. Study Design and Population

The multicenter case–control “IROPICAN” study was launched in 2017–2020 in Iran to assess the association between opium consumption and the risk of four cancer types including the lung, colorectum, bladder, and head and neck cancer [16]. For the current study, we used 4131 participants, including 898 CRC patients and 3233 controls, who were recruited from the main cancer hospitals in 7 provinces (Tehran, Fars, Mazandaran, Kerman, Golestan, Kermanshah, Mashhad) [16]. The inclusion criteria of cases were pathological confirmation with ICD-O-3 codes C18.0–9, colon cancer including proximal colon (from cecum to splenic flexure) and distal colon (from descending colon to sigmoid colon), and with C19–C20 (from the recto-sigmoid junction to the rectum). The controls were enrolled alongside patients among the relatives or friends of patients from non-oncology wards or others who visited the hospital for reasons other than receiving treatment. The controls had to be free of CRC at the date of recruitment. We excluded 60 participants due to failure to complete the Food Frequency Questionnaire (FFQ). The final participants consisted of 865 cases (mean age 57.2) and 3206 controls (mean age 58.5).

### 2.2. Anthropometry and Lifestyle Factor

Details of the study have been described elsewhere [16]. Briefly, trained interviewers collected information such as education, smoking, opium use, socioeconomic status (SES), physical activity, and medical history through face–face interviews. We measured the standing height of the participants and the weight of the control group at the time of enrollment. In contrast, all cases were asked about their body weight before their cancer diagnosis. The body mass index (BMI) and physical activity workload (PPWL), and the socioeconomic status (SES) index were calculated [17,18,19].

### 2.3. Dietary Factors

Dietary habits were assessed with a qualitatively validated Persian Cohort FFQ [20] administered by trained interviewers. The usual consumption of 131 food items, divided into food groups such as bread and cereals, meat, vegetables and fruits, dairy products, oils, sugars, spices, and others, was collected in the last year before cancer diagnosis between cases and before the interviews between controls. In order to calculate daily intake (grams per day per food item), all types of collected consumption frequencies (daily, weekly, monthly, or yearly) were converted to daily frequency and multiplied by the standard portion size (grams) using household measures. Finally, the macro- and micro-nutrient composition was identified using the food composition table (FCT) developed for the Iranian population [21]. This FCT is prepared based on the food compositions provided by the US Department of Agriculture (USDA) [22,23,24,25], and food composition tables prepared in the Near-East [26], and Bahrain [27].

For the purpose of this analysis, we developed an additional food composition database to assess the total choline (mg/day) and selected choline-containing compounds, including the free choline (mg/day), GPC (mg/day), Pcho (mg/day), PtdCho (mg/day), and SM (mg/day) content of each food and ingredient considered in our FFQ, based on available food composition tables [13,28,29,30]. Data matching was performed based on a defined priority order (e.g., similarity of food name, description, macronutrient composition, water content) according to international guidelines [31]. Finally, for each subject we calculated the daily intake of these nutrients based on their information from FFQ (frequency and portion size of food items intake).

### 2.4. Statistical Analyses

The normality of dietary variables was tested by comparing a histogram of the values in this data to a normal probability curve. We used the residual method to adjust the dietary choline intake for total energy by regressing the total and different types of choline intakes on the total energy intake. To evaluate the correlations among the selected choline compounds and betaine, we calculated Spearman correlation coefficients between these energy-adjusted nutrients among the controls. Multivariable-adjusted logistic regression models were used to compute odds ratios (ORs) and 95% confidence intervals (CIs) of CRC (and its different sub-sites) for choline intake (as a continuous variable). The regression models included gender, age (continuous), BMI (continuous), height (continuous), tobacco (smoking and water pipe) consumption (never/ever), opium use (no user/irregular users/regular users), aspirin use (yes/no), province, SES (low/medium/high), physical activity workload (light/moderate/heavy), and intake of vegetables (continuous, g/day), red meat (continuous, g/day), calcium (continuous, g/day), folate (continuous, g/day), and energy (continuous, Kcal/day) as covariates. The participants with missing data for physical activity (24.93%) were coded in a distinct category during the analysis. To consider possible reciprocal confounding between choline subtypes, we also considered additional models adjusted for the other choline subtypes. Based on the distribution of choline intake among the controls, categorical analyses were also conducted using quartiles. Additionally, we performed stratified analyses by gender. The likelihood ratio test was used to calculate the *p*-value for heterogeneity. A *p*-value less than 0.05 was considered statistically significant. The statistical analysis was performed with the software Stata 14 (Stata Statistical Software: Release 14. College Station, TX, USA: Stata Corp LLC).

## 3. Results

Table 1 shows the distribution of socio-demographic characteristics and dietary intake information between cases and controls. Among the 865 CRC cases, 434 were from the colon (including 145 proximal colon cases, 185 distal colon cases, and 104 had overlapping lesions) and 404 were from the rectum. In total, 27 of the cases were from unknown sub-sites. This population was used in previous analyses [21,32]. Around 76% of the CRC cases (671) were older than 50 at the time of diagnosis. Over 50% of the CRC cases were from Fars and Tehran. The controls were more likely to be males (68.71%).

Table 2 presents the means of choline and betaine intakes and the correlation coefficients between each pair of energy-adjusted intakes of selected choline types and betaine among controls. The mean total choline intake was 335 mg/day. More than half of the total choline intake came from PtdCho. The mean betaine intake was 75 mg/day. The correlation of energy-adjusted betaine with choline intakes was low for all the considered types of choline, while the correlation between pairs of energy-adjusted intakes of choline types was strong between Pcho and free choline (Spearman coefficient = 0.81) and between SM and PtdCho (Spearman coefficient = 0.86).

Table 3 shows dietary estimates of choline and betaine and odds ratios (OR) and corresponding 95% confidence intervals (CI) of risk of colorectal cancer according to quartiles as compared to the lowest quartile (ref; Q1) of energy-adjusted choline and betaine intakes. We observed a positive association between the total choline intake and CRC risk (OR = 1.23, 95% CI 1.13–1.33: *p* for trend <0.001). We also found a positive association between specific forms of choline with CRC, including GPC (OR = 1.13, 95% CI 1.00–1.27; *p* for trend = 0.03) and SM (OR = 1.14, 95% CI 1.01–1.28; *p* for trend = 0.02). The dietary intake of betaine had an inverse association with CRC risk (OR = 0.91, 95% CI 0.83–0.99; *p* for trend = 0.03). There was no association between free choline, Pcho, PtdCho, and CRC risk.

The analysis by CRC sub-site revealed a positive association between the total choline and all anatomical locations of tumors. Table 4 reports the results for those types of choline with an association with the overall CRC risk. There were also positive associations between GPC and rectal cancer (OR = 1.18, 95% CI 1.00–1.39) and between SM and colon cancer (OR = 1.21, 95% CI 1.03–1.41). An inverse association was observed between betaine intake and colon cancer (OR = 0.89, 95% CI 0.79–0.99) (Table 4).

Analyses stratified by gender showed that the association between SM and CRC was present among men (OR = 1.20, 95% CI 1.03–1.40) but not women (OR = 1.03, 95% CI 0.85–1.25) (*p*-value for heterogeneity = 0.8073). Conversely, the inverse association between betaine intake and CRC was confirmed among women (OR = 0.84, 95% CI 0.73–0.97) but not men [OR = 0.95, 95% CI 0.85–1.06] (*p*-values for heterogeneity = 0.8625). No interaction was detected between choline species and other risk factors, including opium use and cigarette and waterpipe smoking.

## 4. Discussion

This study provides novel information on the association between the intake of choline, choline-containing compounds, and betaine and the risk of CRC in an Iranian population. Our results showed an increased risk of CRC in subjects reporting a high versus low intake of total choline, GPC, and SM, with associations present for all CRC sub-sites. We also observed an inverse association between betaine intake and CRC, particularly in colon cancer.

Our results were not in line with a case–control study from China that reported an inverse association between high choline intake from PtdCho, GPC, but not Pcho and free choline, and CRC risk, but observed an inverse association between betaine and CRC [14]. Similar to our results, an analysis of the Nurses’ Health Study reported that increasing choline intake was associated with an elevated risk of colorectal adenoma in women, particularly from SM and PtdCho, and betaine was found to be inversely related to colorectal adenoma [33]. A study in men reported no association between CRC and free choline, GPC, Pcho, PtdCho, or SM, and betaine [15]. Conversely, a recent case–control study from Italy reported an inverse association between the total choline intake and risk of CRC and a null relationship between SM and CRC [28]. Several studies also reported inconsistent results on plasma concentrations of choline and its metabolites on CRC [34,35,36]

The inconsistent results of choline and CRC could be explained by different dietary sources and contributions of various choline-containing compounds and other factors, such as tumor location, gender, age, and race. Choline is considered an essential dietary nutrient for humans which can be obtained through endogenous synthesis and from the diet [13,37], but it appears that endogenous synthesis is not sufficient [38]. Dietary sources of choline include eggs, beef, pork, liver, soybean, and wheat germ [9], whereas dairy products appear to be major source of SM, followed by meat, fish, and eggs [39]. As a result, it is likely that the proportion of sources of each nutrient will influence the results. In our population, we found that eggs, wheat bread, dairy products, chicken, beef, lamb meat, and some vegetables such as tomatoes and onions are the main sources of choline. The majority of SM is also found in eggs, chicken, beef, lamb, and dairy products (Appendix A). Although we adjusted our models for potential confounding factors of colorectal cancer, several possible influences in food may affect the results. For example, the reasons for the difference between the findings shown by Lu et al. [14] and our results may be due to different varieties of dietary pattern intakes and food item sources, with more emphasis on vegetable sources in the Chinese population and animal and fatty sources in the Iranian population. Regarding this issue, we repeated our analysis of the association between SM intake and CRC risk after stratification by the total fat intake level and found a higher OR in the highest quartile of fat intake (OR 1.32, 95% CI 1.08–1.61) compared to the lower quartiles [Q1: OR 1.19, 95% CI 0.89–1.58; Q2: OR 0.92; 95% CI 0.70–1.21); Q3: OR 1.04, 95% CI 0.79–1.37)]. In foods, choline is found as water-soluble (free choline, Pcho, and GPC) and as a component of lipids as lipid-soluble forms [13]. Choline is used as the precursor for the synthesis of important molecules through four pathways including (1) as the neurotransmitter where acetylcholine affects the central and peripheral nervous systems, (2) in the large intestine where choline is metabolized to TMA by the gut microbiota before absorption, (3) choline can be irreversibly oxidized to yield betaine which is an important osmolyte and a methyl group donor, and (4) choline is a precursor for the synthesis of phosphatidylcholine, the most abundant form of phospholipid, SM, and other phospholipids in the body [8]. Therefore, choline is involved in a broad range of critical physiological functions across all stages of the life cycle and structural lipoproteins and membrane lipids [40]. Alterations in choline phospholipid metabolism have been demonstrated in various cancers, including colorectal, breast, prostate, and brain [34,41,42,43]. Choline as a source of one-carbon units through its metabolism to betaine and its subsequent effect on homocysteine metabolism has also been linked to cancer [44].

Betaine is obtained from endogenous synthesis via choline metabolism and also from diet sources such as wheat bran, wheat germ, and spinach [45,46]. In our population, the primary diet sources of betaine included wheat bread and grains, tea, and green leafy vegetables (Appendix A). According to previous studies, betaine may exert a protective effect on a variety of cancers, including colorectal cancer [12,34]. Following previous reports, we also found a significant reverse association between betaine intake and CRC risk, particularly in the colon. These results may be explained by its physiological activities in several useful mechanisms in the human body such as anti-inflammatory and anti-oxidative stress activities, reducing homocysteine levels, inhibition of nuclear factor-κB activity, regulation of energy metabolism, and apoptosis [13,39].

Among the strengths of this study is the ability to examine the association between dietary choline types and betaine and CRC risk. Due to limitations in calculating the amounts of different choline compounds, only a few studies have reported these associations. The first database on total choline and its individual forms was made available in 2004 by the USDA and then updated and expanded in 2008 [9,10,47]. These databases include values for free choline, Pcho, GPC, PtdCho, and SM, as well as values for total choline and betaine. Thus, to our knowledge, this is the first large case–control study addressing this topic in the Eastern Mediterranean region. Moreover, we accounted for several confounders in the analyses to reduce the risk of this bias.

Concerning possible limitations, the FFQ dietary estimation method is prone to recall bias since individuals are asked to report their intake retrospectively and usually for prolonged periods (a single past year before diagnosis in the case groups and before the interview in the control groups). Due to circumstances such as lifestyle changes, we were not able to directly estimate the history of diet. Furthermore, participants can be impeded by deliberately misreporting their consumption of certain foods, which may be affected by their characteristics (e.g., age, gender, overweight, or obesity) and cause a differential misclassification of the data with unpredictable effects on the estimated associations. Other misclassifications in the assessment of diet through FFQs can be introduced via the use of food composition databases to calculate different nutrients, particularly to calculate traditional foods that there are not the same food items in different FCT.

## 5. Conclusions

In conclusion, we found that high total intake of choline, GPC, and SM was associated with an elevated risk of CRC. Conversely, high betaine intake was associated with a decreased risk of CRC, as also supported by previous studies. Consequently, it is possible to focus on the intake of betaine sources and manage the use of animal products as references for SM or other choline types with risks of CRC.

## Figures and Tables

**Table 1:** Selected baseline socio-demographic and lifestyle characteristics and dietary intakes of the study participants (IROPICAN study). This population was used in previous analyses [21,32].

	Controls	Cases		
		Colorectal *	Colon	Rectum
**Overall**	3206	865	434	404
**Age, years, N (%)**				
<30	21 (0.66%)	8 (0.92%)	3 (0.69%)	5 (1.24%)
≥30 & <40	227 (7.08%)	60 (6.94%)	32 (7.37%)	27 (6.68%)
≥40 & <50	503 (15.69%)	126 (14.57%)	64 (14.75%)	58 (14.36%)
≥50 & <60	993 (30.97%)	242 (27.98%)	112 (25.81%)	123 (30.45%)
≥60 & <70	1020 (31.82%)	258 (29.83%)	137 (31.57%)	112 (27.72%)
≥70	442 (13.79%)	171 (19.77%)	86 (19.82%)	79 (19.55%)
**Gender, N (%)**				
Women	1003 (31.28%)	368 (42.54%)	193 (44.47%)	169 (41.83%)
Men	2203 (68.71%)	497 (57.46%)	241 (55.53%)	235 (58.17%)
**Province, N (%)**				
Tehran	816 (25.45%)	165 (19.08%)	101 (23.27%)	64 (15.84%)
Fars	943 (29.41%)	248 (28.67%)	93 (21.43%)	155(38.37%)
Kerman	525 (16.38%)	100 (11.56%)	49 (11.29%)	51 (12.62%)
Golestan	373 (11.63%)	149 (17.23%)	89 (20.51%)	53(13.12%)
Mazandaran	136 (4.24%)	59 (6.82%)	34 (7.83%)	25 (6.19%)
Kermanshah	251 (7.83%)	68 (7.86%)	31(7.14%)	35 (8.66%)
Mashhad	162 (5.05%)	76 (8.79%)	37(8.53%)	21 (5.20%)
**BMI, kg/m^2^, mean (±SD)**	26.59 (±4.72)	26.93 (±4.99)	26.91 (±5.07)	26.83 (±4.85)
**BMI, N (%)**				
Underweight (<18.5)	90 (2.81%)	28 (3.24%)	14 (3.23%)	14 (3.47%)
Normal weight (≥18.5 & <25)	1121 (34.97%)	261 (30.17%)	135 (31.11%)	119 (29.46%)
Overweight (≥25 & <30)	1311 (40.89%)	371 (42.89%)	184 (42.40%)	177 (43.81%)
Obese (≥30)	684 (21.33%)	205 (23.70%)	101 (23.27%)	94 (23.27%)
**Work-related physical activity, N (%)**				
Sedentary	1034 (32.27%)	287 (33.18%)	147 (33.87%)	132 (32.67%)
Moderate	701 (21.88%)	155 (17.92%)	78 (17.97%)	72 (17.82%)
Heavy	694 (21.66%)	184 (21.27%)	87 (20.05%)	87 (21.53%)
Unknown	775 (24.19%)	239 (27.63%)	122 (28.11%)	113 (27.97%)
**SES, N (%)**				
Low	861 (26.86%)	337 (38.27%)	159 (36.64%)	161 (39.85%)
Moderate	1078 (33.62%)	234 (27.05%)	118 (27.19%)	109 (26.98%)
High	1267 (39.52%)	300 (34.68%)	157 (36.18%)	134 (33.17%)
**Tobacco consumption, N (%)**				
Never user	2153 (67.16%)	629 (72.72%)	334 (76.96%)	274 (67.82%)
Ever user	1053 (32.84%)	236 (27.28%)	100 (23.04%)	130 (32.18%)
**Opium consumption, N (%)**				
No user	2646 (82.53%)	731 (84.51%)	369 (85.02%)	340 (84.16%)
Regular user	432 (13.47%)	88 (10.17%)	40 (9.22%)	46 (11.39%)
Irregular user	128 (3.99%)	46 (5.32%)	25 (5.76%)	18 (4.46%)
**Aspirin use, N (%)**				
No	2469 (77%)	709 (81.97%)	358 (82.49%)	327 (80.94%)
Yes	737 (22.99%)	156 (18.03%)	76 (17.51%)	77 (19.06%)
**Family History, N (%)**				
No	2534 (79.04%)	653 (75.49%)	330 (76.04%)	304 (75.25%)
Yes	672 (20.96%)	212 (24.51%)	104 (23.96%)	100 (24.75%)
**Dietary items intake, Mean (SD)**				
Total Vegetable (g/day)	481.07 (5.01)	475.35 (8.60)	479.04 (12.69)	473.54 (12.12)
Fiber (g/day)	24.72 (±11.2)	25.86 (±12.4)	25.28 (±12.2)	26.34 (±12.73)
Red Meat(g/day)	16.67 (±17.3)	22.13 (±25.1)	21.79 (±23.3)	22.16 (±26.82)
Folate (mcg/day)	369.97 (2.83)	391.70 (6.32)	388.55 (8.95)	392.63 (9.31)
Calcium (mg/day)	860.35 (±6.6)	908.28 (±14.7)	919.66 (±20.7)	880.24 (±21.2)
Total energy (kcal/day)	2319.45 (±878.1)	2405.61 (±1076)	2387.21 (±1081.9)	2393.21(±1066.3)

* Includes 27 cancers with unknown sub-site; SES: Socio-economic status; BMI: body mass index.

**Table 2:** The mean (SD) intake (mg/day) of choline and betaine intakes and correlation coefficients between energy-adjusted intakes of pairs of selected choline types or betaine in controls. IROPICAN study.

	Total	Controls	Total Choline	Free Choline	GPC	Pcho	PtdCho	SM	Betaine
**Total choline**	334.80 (151.46)	329.17 (146.61)	1.00	0.43 *	0.34 *	0.35 *	0.57 *	0.56 *	0.03 **
**Free choline**	72.06 (32.33)	71.10 (31.51)		1.00	0.62 *	0.81 *	0.50 *	0.53 *	0.10 *
**GPC**	44.01 (23.05)	43.03 (22.24)			1.00	0.72 *	0.34 *	0.58 *	0.02
**Pcho**	16.18 (7.08)	15.96 (6.90)				1.00	0.33 *	0.44 *	0.06 *
**PtdCho**	135.83 (79.66)	132.77 (77.81)					1.00	0.86 *	0.06 *
**SM**	16.15 (8.87)	15.78 (8.69)						1.00	0.02
**Betaine**	74.84 (49.92)	74.94 (48.39)							1.00

* Correlations are significant (*p* < 0.01); ** Correlations are significant (*p* < 0.05); phosphocholine (Pcho), glycerophosphocholine (GPC), phosphatidylcholine (PtdCho), and sphingomyelin (SM).

**Table 3:** Dietary estimates of choline and betaine and odds ratios (OR) and corresponding 95% confidence intervals (CI) of colorectal cancer according to quartiles as compared to the lowest quartile (ref; Q1) of energy-adjusted choline and betaine intakes based on the IROPICAN study in Iran.

Choline Type	Q2		Q3		Q4		OR (95% CI)	*p*-Trend
	Mean (Cut-Off) mg/day	OR (95% CI)	Mean (Cut-Off) mg/day	OR (95% CI)	Mean (Cut-Off) mg/day	OR (95% CI)		
**Total Choline**	288.9 (61–642)	1.02 (0.80–1.32)	303.3 (99–722)	1.53 (1.20–1.95)	470.2 (196–1167)	1.74 (1.36–2.23)	1.23 (1.13–1.33)	<0.001
**Free Choline**	55.5 (13–173)	0.85 (0.65–1.13)	71.9 (26–160)	0.83 (0.60–1.15)	104.1 (47–250)	1.18 (0.81–1.73)	1.0 (0.93–1.19)	0.40
**GPC**	33.9 (8–90)	1.24 (0.95–1.61)	45.3 (21–101)	1.33 (0.97–1.81)	70.4 (34–236)	1.52 (1.05–2.21)	1.13 (1.00–1.27)	0.03
**Pcho**	12.8 (5–35)	1.07 (0.80–1.45)	16.5 (9–31)	0.88 (0.60–1.29)	24.3 (13–61)	0.92 (0.57–1.48)	0.94 (0.80–1.10)	0.464
**PtdCho**	102.3 (15–226)	0.97 (0.73–1.27)	122.8 (38–368)	1.01 (0.75–1.37)	223.5 (78–600)	1.08 (0.75–1.54)	1.03 (0.92–1.15)	0.559
**SM**	12.5 (2–30)	1.06 (0.80–1.40)	15.5 (6–40)	1.30 (0.95–1.79)	25.2 (9–69)	1.45 (1.00–2.09)	1.14 (1.01–1.28)	0.02
**Betaine**	47.3 (7–176)	0.99 (0.78–1.24)	73 (24–147)	0.84 (0.66–1.07)	144.1 (63–602)	0.80 (0.61–1.06)	0.91 (0.83–0.99)	0.03

phosphocholine (Pcho), glycerophosphocholine (GPC), phosphatidylcholine (PtdCho), and sphingomyelin (SM). Adjusted by province, age, SES, gender, BMI, height, tobacco use, family history, opium use, aspirin use, physical activity, red meat intake, vegetable intake, calcium, folate, energy intake.

**Table 4:** Dietary estimates of choline and betaine and odds ratios (OR) and corresponding 95% confidence intervals (CI) of colorectal cancer sub-site according to quartiles as compared to the lowest quartile (ref; Q1) of energy-adjusted choline and betaine intakes based on the IROPICAN study in Iran.

Choline Type	Cancer site	Q2		Q3		Q4		OR (95% CI)	*p*-Trend
		Mean mg/day	OR (95% CI)	Mean mg/day	OR (95% CI)	Mean mg/day	OR (95% CI)		
**Total choline**	**Colon**	287.8	0.99 (0.70–1.41)	308.8	1.63 (1.18–2.26)	471.2	1.76 (1.26–2.45)	1.24 (1.12–1.38)	<0.001
	**Proximal colon**	287.6	1.12 (0.63–2.00)	327.2	1.47 (0.84–2.56)	431.2	2.11 (1.23–3.63)	1.30 (1.09–1.54)	0.003
	**Distal colon**	300.6	0.94 (0.57–1.57)	305.6	1.77 (1.11–2.82)	465.9	1.41 (0.86–2.30)	1.17 (1.00–1.36)	0.04
	**Rectum**	285.1	1.11 (0.79–1.57)	293.1	1.52 (1.09–2.13)	468.6	1.87 (1.34–2.61)	1.24 (1.12–1.38)	<0.001
**GPC**	**Colon**	33.5	1.10 (0.76–1.57)	44.7	1.08 (0.71–1.64)	70.3	1.34 (0.82–2.19)	1.08 (0.92–1.27	0.298
	**Proximal colon**	33.6	0.76 (0.41–1.38)	45.45	0.73 (0.36–1.45)	66.1	1.06 (0.47–2.37)	1.01 (0.78–1.32)	0.902
	**Distal colon**	34.9	1.32 (0.79–2.18)	44.1	1.00 (0.55–1.83)	68.7	1.02 (0.49–2.08)	0.96 (0.76–1.20)	0.744
	**Rectum**	33.7	1.28 (0.89–1.84)	45.1	1.58 (1.04–2.40)	69.9	1.67 (1.00–2.78)	1.18 (1.00–1.39)	0.04
**SM**	**Colon**	12.8	1.18 (0.80–1.73)	15.1	1.57 (1.02–2.42)	25.8	1.73 (1.05–2.83)	1.21 (1.03–1.41)	0.01
	**Proximal colon**	13.9	0.84 (0.44–1.61)	15.6	1.38 (0.69–2.76)	25.3	1.15 (0.51–2.61)	1.10 (0.85–1.42)	0.442
	**Distal colon**	11.6	1.09 (0.62–1.92)	15.3	1.56 (0.85 2.86)	25.2	1.83 (0.91–3.68)	1.23 (0.98–1.54)	0.063
	**Rectum**	12.1	0.90 (0.61–1.32)	15.8	0.98 (0.64–1.51)	24.3	1.08 (0.66–1.78)	1.03 (0.88–1.21)	0.687
**Betaine**	**Colon**	47.1	0.98 (0.72–1.32)	72.1	0.80 (0.58–1.11)	156.9	0.74 (0.51–1.07)	0.89 (0.79–0.99)	0.04
	**Proximal colon**	52.3	0.82 (0.50–1.35)	69	0.97 (0.58–1.61)	141.8	0.66 (0.35–1.24)	0.89 (0.74–1.08)	0.260
	**Distal colon**	44.6	1.02 (0.66–1.57)	72.6	0.65 (0.39–1.06)	161.5	0.76 (0.45–1.30)	0.88 (0.74–1.04)	0.140
	**Rectum**	47.1	0.94 (0.68–1.30)	73.7	0.83 (0.60–1.16)	131.8	0.75 (0.51–1.08)	0.89 (0.79–1.00)	0.069

Phosphocholine (Pcho), glycerophosphocholine (GPC), phosphatidylcholine (PtdCho), and sphingomyelin (SM). Adjusted by province, age, SES, gender, BMI, height, tobacco use, family history, opium use, aspirin use, physical activity, red meat intake, vegetable intake, calcium, folate, energy intake.

**Figure A1:**
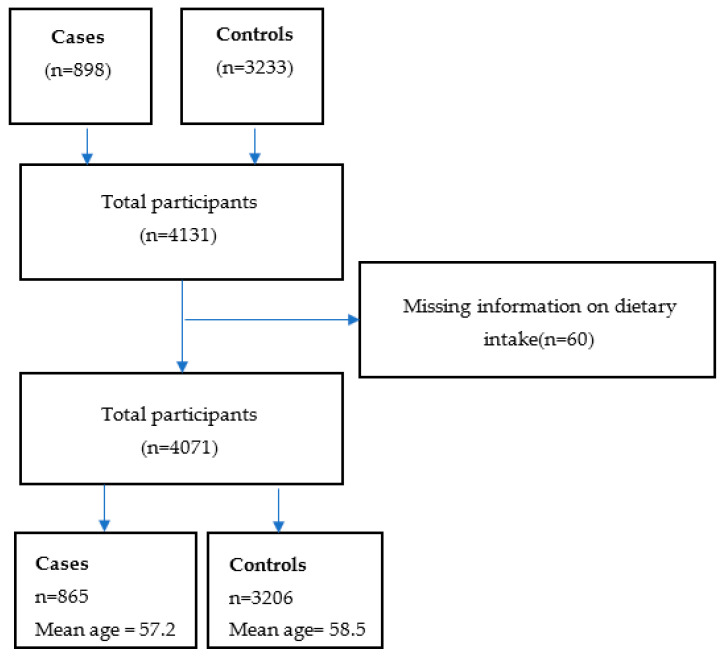
Flow diagram for the inclusion and exclusion of criteria for participants in the case–control study.

## Data Availability

The data of this study are available from the corresponding author upon reasonable request.

## Abbreviations

BMI	The body mass index
CRC	Colorectal cancer
CI	Confidence interval
FCT	Food composition table
FINJEM	Finland Job Exposure Matrix
FFQ	Food Frequency Questionnaire
GPC	Glycerophosphocholine
ORs	Odds ratios
PtdCho	Phosphatidylcholine
PPWL	Physical activity workload
Pcho	Phosphocholine
SM	Sphingomyelin
SD	Standard deviations
SES	Socioeconomic status
TMA	Trimethylamine
USDA	US Department of Agriculture

## Appendix A

The top ten main sources of selected choline compounds and betaine intake among controls.

**Table A1:** The Top Ten Main dietary Sources of Selected Choline Compounds and Betaine Intake among Controls.

List of Choline Type	Source 1	Source 2	Source 3	Source 4	Source 5
Total choline	Eggs	Yogurt	Bread	Chicken	Lamb products and red meat
Free choline	Onion	Tomatoes	Lamb products and red meat	Tea	Milk
GPC	Yogurt	Milk	Doogh	Lamb products and red meat	Rice
Pcho	Yogurt	Tomatoes	Milk	Onion	Chicken
PtdCho	Eggs	Chicken	Lamb products and red meat	Apples	Lentil, Mung, Bean
SM	Chicken	Eggs	Yogurt	Lamb products and red meat	Cheese
Betaine	Bread	Pasta and noodles	Tea	Beets, turnips	Green leafy vegetables

phosphocholine (Pcho), glycerophosphocholine (GPC), phosphatidylcholine (PtdCho), and sphingomyelin (SM).
